# Combined treatment of rapamycin and dietary restriction has a larger effect on the transcriptome and metabolome of liver

**DOI:** 10.1111/acel.12175

**Published:** 2013-12-05

**Authors:** Wilson C Fok, Alex Bokov, Jonathan Gelfond, Zhen Yu, Yiqiang Zhang, Mark Doderer, Yidong Chen, Martin Javors, William H Wood, Yongqing Zhang, Kevin G Becker, Arlan Richardson, Viviana I Pérez

**Affiliations:** 1 Department of Cellular and Structural Biology, The University of Texas Health Science Center at San AntonioSan Antonio, TX, 78229, USA; 2 Barshop Institute for Longevity and Aging Studies, The University of Texas Health Science Center at San AntonioSan Antonio, TX, 78229, USA; 3 Department of Epidemiology & Biostatistics, The University of Texas Health Science Center at San AntonioSan Antonio, TX, 78229, USA; 4 Linus Pauling Institute, Oregon State UniversityCorvallis, OR, 97331, USA; 5 Department of Physiology, The University of Texas Health Science Center at San AntonioSan Antonio, TX, 78229, USA; 6 Greehey Children’s Cancer Research Institute, The University of Texas Health Science Center at San AntonioSan Antonio, TX, 78229, USA; 7 Cancer Therapy and Research Center, The University of Texas Health Science Center at San AntonioSan Antonio, TX, 78229, USA; 8 Department of Psychiatry, The University of Texas Health Science Center at San AntonioSan Antonio, TX, 78229, USA; 9 National Institute on AgingBaltimore, MD, 21224, USA; 10 Research Service, Audie Murphy VA Hospital (STVHCS)San Antonio, TX, 78229, USA; 11 Department of Biochemistry and Biophysics, Oregon State UniversityCorvallis, OR, 97331, USA

**Keywords:** dietary restriction, metabolome, rapamycin, transcriptome

## Abstract

Rapamycin (Rapa) and dietary restriction (DR) have consistently been shown to increase lifespan. To investigate whether Rapa and DR affect similar pathways in mice, we compared the effects of feeding mice *ad libitum* (AL), Rapa, DR, or a combination of Rapa and DR (Rapa + DR) on the transcriptome and metabolome of the liver. The principal component analysis shows that Rapa and DR are distinct groups. Over 2500 genes are significantly changed with either Rapa or DR when compared with mice fed AL; more than 80% are unique to DR or Rapa. A similar observation was made when genes were grouped into pathways; two-thirds of the pathways were uniquely changed by DR or Rapa. The metabolome shows an even greater difference between Rapa and DR; no metabolites in Rapa-treated mice were changed significantly from AL mice, whereas 173 metabolites were changed in the DR mice. Interestingly, the number of genes significantly changed by Rapa + DR when compared with AL is twice as large as the number of genes significantly altered by either DR or Rapa alone. In summary, the global effects of DR or Rapa on the liver are quite different and a combination of Rapa and DR results in alterations in a large number of genes and metabolites that are not significantly changed by either manipulation alone, suggesting that a combination of DR and Rapa would be more effective in extending longevity than either treatment alone.

## Introduction

Rapamycin (Rapa) is a macrocyclic lactone produced by the bacterium *Streptomyces hygroscopicus* isolated from soil samples from Easter Island. Initially, Rapa was developed as an antifungal agent; however, when it was discovered that Rapa had antirejection properties without the side effects associated with other antirejection agents, it was approved by the FDA to prevent the rejection of organs in transplant patients in combination with other immunosuppressive agents (Camardo, 2003). In 1994, three groups showed that Rapa bound a specific protein, Target of Rapamycin (TOR; Brown *et al*., 1994; Cafferkey *et al*., 1994; Sabatini *et al*. 1994), which subsequently was found to be a serine/threonine kinase that is the regulatory nexus in the response of eukaryote cells to nutrients, growth factors, and cellular energy status. In mammals, TOR (mTOR) forms two major complexes: mTORC1, which is inhibited by rapamycin (Sarbassov *et al*., 2006) and mTORC2, which has been reported to be insensitive to rapamycin; however, recent data suggest that long-term rapamycin treatment might inhibit mTORC2 (Thomson *et al*., 2009; Lamming *et al*., 2012). The mTORC1 consists of mTOR, Raptor, mLST8, FKBP38, PRAS40, and Deptor. Rapa inhibits TOR by binding to FKBP12, which then binds to the c-Terminal region of TOR disrupting TOR activity (Hay & Sonenberg, 2004).

In 2009, Harrison *et al*. showed that Rapa extended the lifespan of both male and female mice when initiated at 19 months of age. This was the first rigorously performed demonstration that a pharmaceutical intervention consistently increases longevity in a mammal. Since the initial report, two other studies have shown that Rapa increases the lifespan of mice. Miller *et al*. (2011) showed that Rapa increased the lifespan of male and female mice when initiated at 9 months of age, and Anisimov *et al*. (2011) reported that Rapa increased the lifespan of female mice when administrated intermittently (2 weeks per month) starting at 2 months of age. Rapa also has been shown to increase the chronological lifespan of yeast (Powers *et al*., 2006) and the lifespan of *Drosophila* (Bjedov *et al*., 2010). Thus, the effect of Rapa on the lifespan of mice is reproducible and appears to be evolutionarily conserved.

Because Rapa inhibits the action of mTOR, the major nutrient sensing pathway in mammals, it was initially proposed that Rapa’s mechanism of action was similar to that of dietary restriction (DR; Kaeberlein & Kennedy, 2009), in which animals are fed 30–50% less than the amount consumed by mice fed *ad libitum* (AL). DR is the most studied manipulation known to extend lifespan and delay aging in rodents and invertebrates. To test the hypothesis that DR and Rapa affect lifespan similarly, epistasis studies on lifespan have been done in invertebrate models with DR and genetic manipulations of the TOR pathway. Kaeberlein’s group showed in Saccharomyces *cerevisiae* that a mutant of TOR increased the replicative lifespan in a similar fashion to that observed with DR and that treatment with DR in this mutant showed no further effect on life extension than DR or the TOR mutant alone (Kaeberlein *et al*., 2005). Similarly in *Caenorhabditis elegans*, inhibition of TOR pathway using RNAi showed an increase in lifespan that was not further extended when TOR RNAi was used in an *eat-2* mutant, which was a DR mimetic in *C. elegans* (Hansen *et al*., 2007). These results point to DR and Rapa sharing similar mechanisms in lifespan extension. However, Partridge’s group showed that *Drosophila melanogaster* fed Rapa had an increase in maximum lifespan above the increase in lifespan shown in flies on DR alone (Bjedov *et al*., 2010). The *Drosophila* studies suggest that Rapa may be extending lifespan through pathways partially independent of those used by DR.

In a previous study, we compared the effect of Rapa and DR on various biochemical/physiologic parameters known to be altered by aging in mice (Fok *et al*., 2012). We found that both DR and Rapa showed a decrease in mTOR signaling and an increase in autophagy. However, DR and Rapa differed in their effects on the glutathione redox state and glucose and insulin tolerance. Because our previous study was focused on specific pathways/parameters altered by aging, we sought an unbiased approach to compare the effect of Rapa and DR on the overall physiologic phenotype of mice. Using analyses of the transcriptome and metabolome, we compared the similarities and differences of DR and Rapa on liver isolated from mice fed a Rapa diet, a DR diet, and a combination of Rapa and DR diets (Rapa + DR). Our data show that over 80% of the changes in the transcriptome and metabolome in the liver are unique to either Rapa or DR, i.e., < 20% are shared by DR and Rapa. Interestingly, when mice are fed Rapa + DR, a large number of genes (40%) are observed to change significantly that are not significantly altered by either DR or Rapa alone.

## Results

We first used microarray analysis as an unbiased approach to compare gene expression in the livers of mice fed either AL, DR, Rapa, or Rapa + DR. Because rapamycin is known to inhibit the mTOR signaling pathway, we measured mTOR signaling (ratio of phosphorylated S6 compared to the total S6) in the livers of the four groups of mice (Fig. S1, Supporting information). The Rapa and DR groups showed significant decreases (34% and 60%, respectively, comparing the means of each group) in mTOR signaling when compared with AL, which we have previously observed (Fok *et al*., 2012). We found that the Rapa + DR group had a 68% decrease in mTOR signaling relative to AL; however, mTOR signaling was not significantly different from either the DR or Rapa group. We also measured the Rapa levels in the livers of the Rapa and Rapa + DR groups. The data in Fig. S2 show that Rapa levels are not significantly different in the Rapa and Rapa + DR mice.

**Figure 1:**
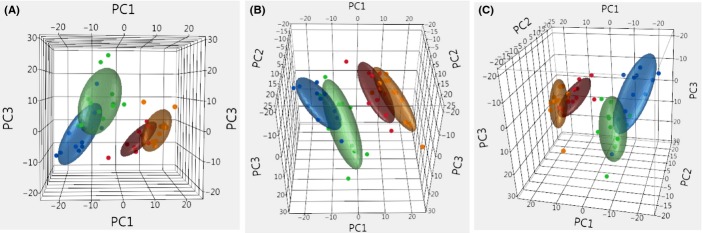
Principal component analysis (PCA) shows the separation of dietary restriction (DR) and Rapa groups. Using all the probes detected (15 444 probes, p-detection < 0.02), the variance in the top three principal components is shown in three orientations (A–C) in 3-D plots. Each dot represents an individual sample in the respected group and the ellipsoid is the general volume of where the samples lie. The color representation for *ad libitum* (AL) is blue, DR is red, Rapa is green, and Rapa + DR is orange. The statistical comparison of the top three principal component was calculated using a linear discriminant predictor which gives an AUC of 0.5 (no separation between groups) to an AUC of 1 (perfect separation) between groups with the 95% confidence interval (Table S1). Figure show that there is a separation of 1 between DR and Rapa or AL, and a separation of 0.8 between Rapa to AL, which is a good separation but not perfect, indicating some overlap in gene expression. Similarly, when we compared DR with Rapa + DR, we found a separation of 0.8. However, when we compared Rapa + DR to either Rapa or AL, we obtained a value of 1.

**Figure 2:**
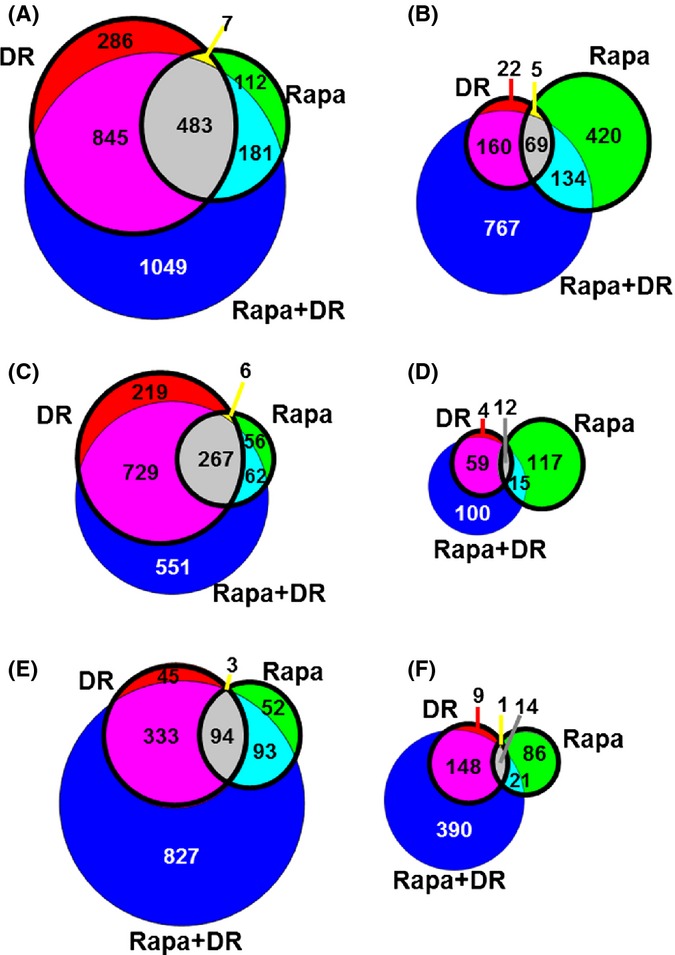
Gene expression analysis shows significant differences between dietary restriction (DR) and Rapa treatment. The number of genes showing a significant difference in DR, Rapa, or Rapa + DR relative to AL was determined. Venn diagrams show the number of genes significantly up-regulated (A) or down-regulated (B) using a filtering criteria of *q* < 0.05 and > 15% change; or genes up-regulated (C) and down-regulated (D) using a criteria of *q* < 0.05 and > 30% change; and genes up-regulated (E) and down-regulated (F) using a criteria of *q* < 0.0001 and > 15% change. The bolded circles indicate the number of genes significantly changed in DR vs. Rapa only. The colors indicate genes significantly changed in each respective bin: red, DR; green, Rapa; blue, Rapa + DR; yellow, shared between DR and Rapa; cyan, shared between Rapa and Rapa + DR; magenta, shared between DR and Rapa + DR; and gray, shared among all three comparisons. AL, *ad libitum*.

The global transcriptomes of the four groups of mice were compared using a principal component analysis (PCA), which allowed an unbiased analysis in a format in which the groups could be visually and quantitatively compared. Figure 1 shows that using the top three principal components, the DR-fed mice appear as a separate group from the AL and Rapa-fed mice; however, they share some overlap with the Rapa + DR mice. Using the linear discrimination predictor (Table S1A, Supporting information) and the quadratic discriminant analysis (Table S1B), we statistically compared the four groups in Fig. 1 and found that the DR and Rapa groups showed a perfect separation. We next identified the genes in the DR, Rapa, and Rapa + DR mice that differed significantly to mice fed AL using a fold change of > 15% and a false discovery rate (FDR) of *q* < 0.05. Because these filtering criteria were not particularly stringent, they allowed us to capture the maximum number of genes that potentially changed between groups. We observed that 1621 genes (84%) were up-regulated by DR and 783 genes (41%) were up-regulated by Rapa (Fig. 2A bolded circle). On the other hand, 256 genes (31%) were down-regulated by DR and 628 genes (77%) were down-regulated by Rapa (Fig. 2B). When we compare DR and Rapa groups only (Fig. 2A,B), we observed that 490 up-regulated genes (26%) were shared by DR and Rapa, and 74 down-regulated genes (9%) were shared by DR and Rapa. Thus, our dataset showed that 74% of up-regulated genes and 91% of down-regulated genes were not shared by DR and Rapa.

Because the similarities between the DR and Rapa groups might be masked by the filtering criteria we employed, we used a more restrictive criteria: > 30% change with *q* < 0.05 and > 15% with *q* < 0.001. The number of transcripts that were found to change significantly was reduced over 50% for both analyses (from 2724 to 1546 and 899 genes for > 30% change, and *q* < 0.001, respectively). Figure 2(C–F) shows that the pattern of changes in gene expression in the DR and Rapa mice were similar to that observed using a cut-off of > 15% change (*q* < 0.05). More importantly, the fraction of genes that were shared by DR and Rapa mice were actually less using the more restrictive criteria (20% for > 30% change with *q* < 0.05, and 16% for > 15% change with *q* < 0.001; Table S2A,B). A list of the genes that showed a change in expression in the four groups and using the three filtering criteria are shown in File S1 tab1 (Supporting information).

In the Rapa + DR mice, we observed that 2558 genes were up-regulated (Fig. 2A) and 1130 genes were down-regulated by Rapa + DR relative to AL (Fig. 2B). Rapa + DR up-regulated 1049 (35%) and down-regulated 767 (49%) genes were not significantly altered by either DR or Rapa alone (Fig. 2A,B). Considering all the groups (DR, Rapa, and Rapa + DR to AL), 483 up-regulated genes (16% of total up-regulated genes) and 69 of down-regulated genes (4% of total down-regulated genes) were shared among all three groups, with DR and Rapa + DR sharing more genes than Rapa and Rapa + DR. For example, the 1328 genes (45%) up-regulated genes were shared by DR and Rapa + DR vs. 664 genes (23%) were shared by Rapa and Rapa + DR. Considering all three groups, the Rapa + DR group had the largest number of genes showing a significant change when compared with AL; the 2558 genes up-regulated by Rapa + DR constituted 86% of all significantly up-regulated genes compared to 55% for DR and 27% for Rapa. Similarly, the 1130 genes down-regulated by Rapa + DR constituted 72% of all significantly down-regulated genes compared to 16% for DR and 40% for Rapa. When we analyzed the genes changed by Rapa + DR relative to DR (Rapa + DR/DR), we observed that only 236 genes were significantly different between the Rapa + DR and DR groups. Similar results were obtained we used the more restrictive filtering criteria (Fig. 2C–F and Table S2C,D).

**Figure 3:**
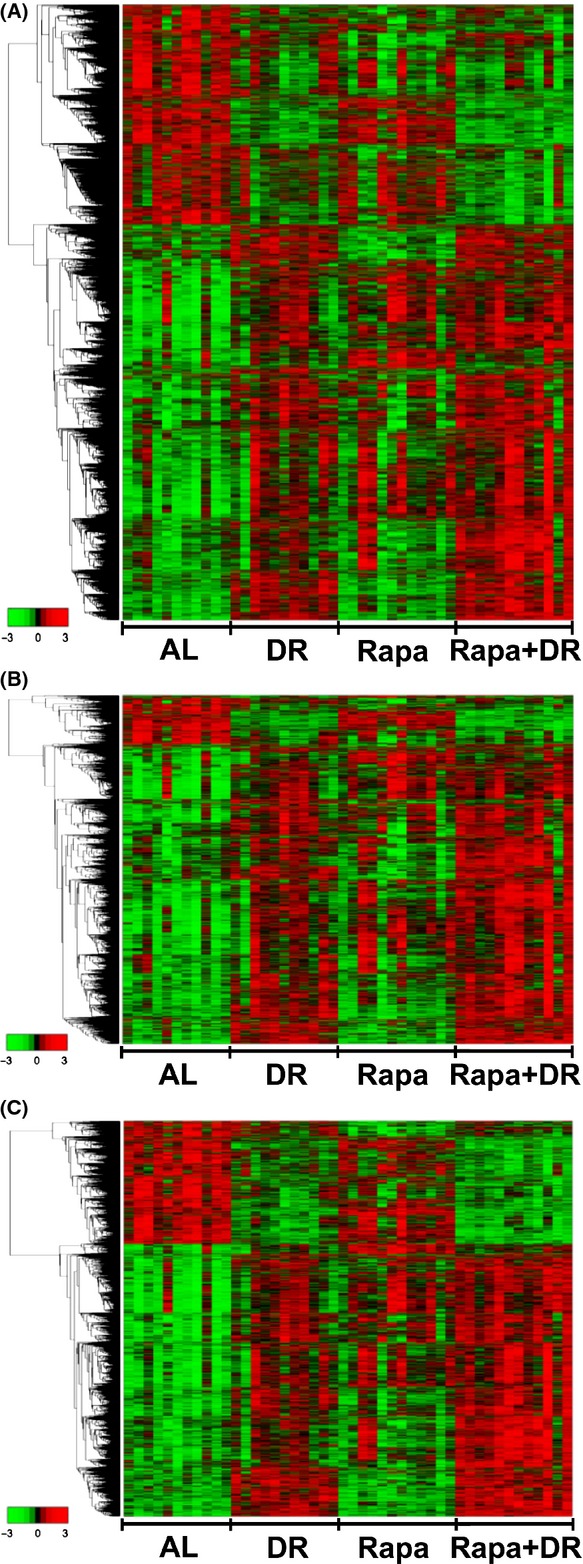
Heatmap Analysis highlight similarities between dietary restriction (DR) and Rapa + DR treatment. The heatmap shows the expression of all the significantly changed probes that showed change relative to AL for DR, Rapa, and Rapa + DR groups clustered using average linkage hierarchical cluster with Euclidean distance using a criteria of *q* < 0.05 and > 15% change (A), *q* < 0.05 and > 30% change (B), and *q* < 0.001 and > 15% change (C). Red indicates high, black indicates middle, and green indicates low level expressing probes. The names of the genes that change significantly are given in File S1 tab1 by the same top to bottom order as on the heatmaps. AL, *ad libitum*.

Figure 3 shows the heatmaps generated for the 4540, 2197, and 2116 transcripts that changed significantly using the filtering criteria. One-fifth to one-third of the transcripts that changed in DR were down-regulated in most of the DR and Rapa + DR animals compared to the AL animals. On the other hand, few of the Rapa animals showed a down-regulation of these transcripts. When compared with AL mice, the number of mice showing an up-regulation of transcripts was greatest for the Rapa + DR mice and least for the Rapa mice. It is interesting to note that the Rapa + DR mice showed a general trend of an increased intensity of over expression, and almost all mice in the group showed up-regulation of the genes.

**Figure 4:**
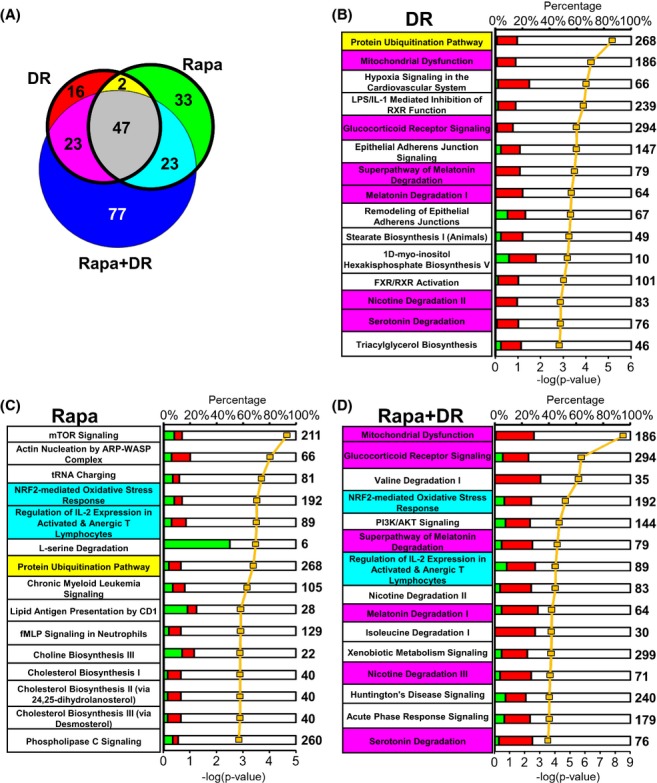
Pathways analysis indicates significant differences between dietary restriction (DR) and Rapa treatment. Pathway analyses were conducted using the genes found to be significantly changed in DR, Rapa, and Rapa + DR relative to AL. The data from the Ingenuity Pathway Analysis (A) is represented by a venn diagram showing the number of pathways that are significantly changed as determined using a Fisher’s exact test of *P* < 0.05. The bolded circles indicate the number of pathways significantly changed for DR vs. Rapa only. The colors indicate significantly changed pathways in each respective bin: red, DR; green, Rapa; blue, Rapa + DR; yellow, shared between DR and Rapa; cyan, shared between Rapa and Rapa + DR; magenta, shared between DR and Rapa + DR; and gray, shared among all three comparisons. The top 15 pathways from Ingenuity Pathway Analysis ranked by the lowest *P*-values as determined by the Fisher’s exact test are shown for DR (B), Rapa (C), and Rapa + DR (D). The yellow line is the -log of the *P*-value and the bolded number on the right side of the graph indicates the total number of possible genes of that pathway. The green color indicates the percentage of the genes from the submitted list that are down-regulated whereas the red color indicates up-regulated genes and white color indicates the percentage not found in the significant gene list. Pathways in the top 15 that are shared between DR and Rapa are highlighted in yellow, whereas pathways shared between Rapa and Rapa + DR are highlighted in magenta, and pathways shared between Rapa and Rapa + DR are highlighted in cyan. No highlights indicate no sharing or overlap with any treatment. File S1 lists the pathways significantly changed in the Ingenuity Pathway Analysis (tab2, tab3, and tab4) and comparisons of significant pathways (tab5). AL, *ad libitum*.

We also used the Ingenuity Pathway Analysis to identify the pathways that were significantly altered when compared with mice fed AL using genes significantly changed in DR, Rapa, or Rapa + DR and a criteria of *q* < 0.05 and ≥ 15%. We found that 88 pathways were changed by DR, and 105 pathways by Rapa (Fig. 4A). Of these pathways, 49 (34%) were shared between DR and Rapa. Figure 4(B,C) show the top 15 pathways ranked by lowest *P*-value in the DR and Rapa groups, respectively. Only one pathway was shared between DR and Rapa in the top 15 pathways. When we added the pathways that were significantly altered by the combined group (Rapa + DR), we observed that 170 pathways were significantly changed by Rapa + DR (Fig. 4A), and 47 pathways (21%) were shared among all three groups (DR, Rapa, and Rapa + DR). Once again the Rapa + DR group showed the largest number of pathways that were significantly changed when compared with the AL group, e.g., 77% (170 pathways) were significantly changed by Rapa + DR, compared to 40% for DR and 48% for Rapa. Moreover, the Rapa + DR group showed a significant change in 77 pathways unique to Rapa + DR, compared to 16 unique for DR and 33 unique for the Rapa (Fig. 4A, blue, red and green bins, respectively). Figure 4(D) shows the top 15 pathways for the Rapa + DR mice. Two pathways were shared between Rapa and Rapa + DR and six pathways were shared between DR and Rapa + DR.

**Figure 5:**
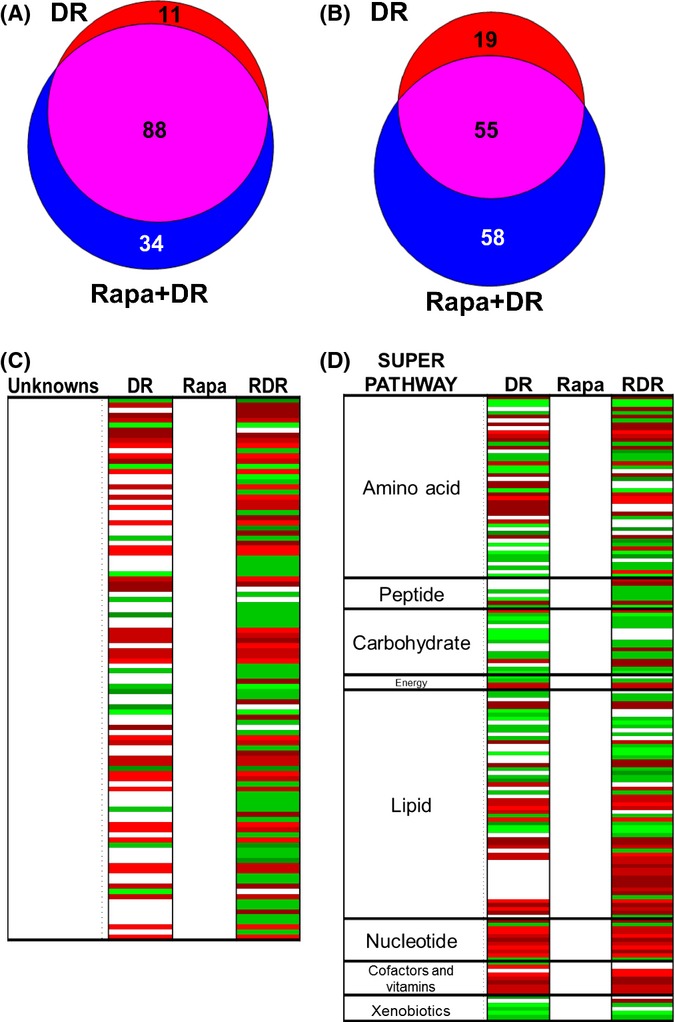
Metabolomic analysis shows significant differences in metabolites between dietary restriction (DR) and Rapa treatment. Using gas or liquid chromatography followed by mass spectrometry, the separation and quantification of small molecules and biochemicals in the liver samples were possible. Metabolomic profiles obtained by Metabolon contained metabolites that were identified based on a library of approximately 1000 purified standards as well as unique compounds that were not in Metabolon’s library, i.e., unknown metabolites. Significantly changed metabolites were found using a filtering criteria of *q* < 0.05 and > 15% change in DR, Rapa, and Rapa + DR relative to *ad libitum* (AL). Venn diagrams show the number of metabolites significantly increased (A) or decreased (B). The bolded circles indicate the number of metabolites significantly changed in DR vs. Rapa only. The colors indicate metabolites significantly changed in each respective bin: red, DR; green, Rapa; blue, Rapa + DR; yellow, shared between DR and Rapa; cyan, shared between Rapa and Rapa + DR; magenta, shared between DR and Rapa + DR; and gray, shared among all three comparisons. (C) Heatmap of unknown metabolites (105) that significantly changed with DR, Rapa, or Rapa + DR relative to AL. (D) Heatmap of known metabolites (157) that significantly changed with DR, Rapa, or Rapa + DR relative to AL are listed by super pathways. Red indicates significantly increased, green indicates significantly decreased, and white indicates metabolites that did not change significantly.

We also used metabolic analysis of the liver tissue from the four groups as another unbiased approach to compare similarities and differences between the groups. Using a filtering criteria of *q* < 0.05 and a change of > 15%, we observed that relative to AL, 99 metabolites were increased and 74 metabolites were decreased by DR. No metabolites were changed significantly with Rapa. When we added the Rapa + DR group into the analysis, we observed that relative to AL, 122 metabolites were increased and 113 metabolites decreased by Rapa + DR. Ninety-two metabolites were altered in the Rapa + DR that were not observed in the DR group; 34 metabolites increased and 58 decreased with respect to the AL group. As can be seen in Fig. 5(A,B), 88 (66%) and 55 (42%) of the metabolites increased and decreased similarly in DR and Rapa + DR. Over 80% of the metabolites that changed with DR were observed to change with Rapa + DR. Of the 92 metabolites significantly changed by Rapa + DR but not by DR, 87% changed in same direction in the DR and Rapa + DR groups.

We also compared the changes in levels of each of the 106 unknown (Fig. 4C) and the 159 known (Fig. 4D, names of these metabolites are given in File S1 tab6) metabolites for the DR and Rapa + DR groups. The profiles for DR and Rapa + DR groups were similar in that the individual metabolites change in the same direction. When comparing the super pathways, we observed that the increase in 44 known metabolites unique to Rapa + DR mice occurred primarily in the lipid and amino acid super pathways.

## Discussion

Because Rapa inhibits the major nutrient signaling pathway in eukaryote cells and because DR limits the dietary intake of an organism, it is logical that investigators initially speculated that rapamycin increased lifespan in mice by ‘mimicking’ the downstream signaling effects of DR without changes in body weight or reduction in food consumption (Kaeberlein & Kennedy, 2009). There were also suggestions that DR and Rapa may be similar because one of the inputs that negatively regulate mTOR signaling is through the availability of amino acids.

The purpose of this study was to use an unbiased analysis of the transcriptome and metabolome to identify similarities and differences in transcriptome and metabolome of DR and Rapa mice. The DR (40%) and Rapa (14 ppm) treatment regimens used in this study have been shown to increase the lifespan of mice (Ikeno *et al*., 2005; Harrison *et al*., 2009). We focused on the liver because liver is the first tissue exposed to rapamycin absorbed in the gut, it is relatively homogenous with hepatocytes constituting 80% of the liver volume (Kmiec, 2001), it expresses a diverse range of metabolic pathways, and it plays a major role in drug metabolism. Over the past two decades, numerous laboratories have studied the effect of DR on liver function, and these studies have shown that DR reduces lipid content (Larson-Meyer *et al*., 2008; Moura *et al*., 2012), improves protein degradation/turnover (Cavallini *et al*., 2001), and mitochondria function (Hagopian *et al*., 2005). Recently, Zhang *et al*. (2013b) showed that DR reduced liver pathology and improved liver function (lower plasma levels of alanine amino transferase and alkaline phosphatase) in *Sod1*^−/−^ mice, which exhibited increased liver pathology. There is also substantial information on the effect of long-term Rapa feeding on liver showing that Rapa either improves or has no effect on liver pathology, i.e., no evidence of toxicity. Wilkinson *et al*. (2012) showed that Rapa significantly reduced liver degeneration in old male mice, and this effect was dose dependent up to levels of Rapa three-fold higher than the dose used in this study. Recently, Zhang *et al*. (2013a) reported that Rapa reduced liver pathology (from 24% in the controls to 7% in Rapa) in old male mice, and Neff *et al*. (2013) reported that Rapa significantly reduced microgranulomas in livers of old male but had no effect on liver hepatic fibrosis.

Using principal component analysis of the transcriptome data we found that the components describing most of the variance in DR and Rapa showed no overlap, demonstrating that the global gene expression profiles of mice treated by DR or Rapa fall-out as distinct groups. We also found that < 20% mRNA transcripts that changed significantly by either DR or Rapa were shared by both DR and Rapa. DR has a greater effect on up-regulated genes, and Rapa has a greater effect on down-regulated genes. It is possible that higher doses of Rapa (over 14 ppm) would generate a transcriptome profile more similar to that observed with DR. As we cannot discount this possibility, our preliminary data with female mice (Fig. S3) show that of the 20 transcripts studied, 18 do not show a change in expression when higher doses of Rapa (e.g., 20, 22, and 42 ppm of Rapa) are given.

When we compared our data to the Dietary Restriction Gene database (GenDR, which contained data from 61 datasets using different animals such as mouse, rat, and pig and from 19 different tissues), we found that 129 of the genes we identified that changed with DR matched the 174 genes currently in the GenDR database (Plank *et al*., 2012). On the other hand, only 13 genes that changed with Rapa-treatment were on the GenDR list.

Our analysis of the genes that were up-regulated by DR but not by Rapa identified genes related to mitochondrial function (e.g., NADH dehydrogenase, ATPase subunits, pyruvate dehydrogenase subunits such as lipoamide, and surfeit genes such as *Surf-1*), suggest that certain aspects of mitochondria function are improved by DR, which is consistent with the data showing that mitochondrial proton leak and H_2_O_2_ production were reduced by DR (Hagopian *et al*., 2005). Several genes for antioxidant enzymes (*Sod-2*, catalase, peroxiredoxin, hemoxigenase) also showed a significant increase only by DR, which was consistent with the numerous reports that DR reduced oxidative damage in liver (Bokov *et al*., 2004). As for the genes that were up-regulated only by Rapa, we found *Credl2*, *Pdia4*, calreticulin, calpain2, and *Sumo-3*, suggesting that Rapa up-regulates the unfolding protein response pathway. On the other hand, DR down-regulates genes of the complement, major urinary proteins (which is consistent with an early observation by Richardson *et al*., 1987), and glucokinase, which is associated with the control of energy expenditure and shifting the metabolism depending on glucose levels (Matschinsky, 2005). As for genes down-regulated by Rapa, we found that transcripts for glutathione-S-transferase (i.e., *Gstm2* and *Gstt3*) were reduced, suggesting that GSH synthesis may be affected by Rapa. Interestingly, both DR and Rapa increased the expression of proteasome subunits genes (Psmd), ubiquitin ligases (*Rbx1*), and heat shock proteins (*Hsp90*) as well as some genes in autophagy pathway, e.g., *Atg12,* suggesting that both DR and Rapa improve protein quality.

Our pathway analysis showed that more than 65% of the pathways identified were unique to either DR or Rapa treatment, which fits with our transcript analysis. For example, if we consider the top 15 pathways ranked by *P*-value, only the protein ubiquitination pathway was shared by DR and Rapa, which agreed with our gene analysis, again suggesting that both DR and Rapa might improve protein quality by removing damaged/misfolded proteins. mTOR and Nrf2 signaling pathways are in the top 15 pathways for Rapa, and as they were not in the top 15 pathways in DR, they were significantly altered by DR. Similarly, as mitochondrial dysfunction and glucocorticoid receptor signaling pathways were not in the top 15 pathways for Rapa treated mice, they were significantly altered by Rapa. The mTOR signaling pathway was the pathway ranked the highest in the Rapa treated mice, which was not surprising because Rapa’s known target was mTOR. The two pathways (within the top 15 pathways) shared by Rapa and Rapa + DR were Nrf2 and regulation of IL-2 pathways. The IL-2 pathway is a key component of the immune response against infections but has been shown to be effective in certain cancers such as metastatic melanoma. The Nrf2 pathway could be an important mechanism in the increased lifespan of the DR and Rapa mice because it plays a major role in protecting organisms against stress, particularly oxidative stress. In addition, Pearson *et al*. (2008), showed that *Nrf2*^−/−^ mice were more sensitive to carcinogen exposure than wild type mice and that deletion of the *Nrf2* gene prevented the ability of DR to suppress tumor formation from carcinogen exposure. These findings suggest that alteration in the Nrf2 pathway by DR and Rapa might play a critical role in the reduction of cancer observed in with DR and Rapa (Sharp & Richardson, 2011).

One of the most interesting outcomes of our study was the major increase in the number of genes (more than 1800) that were significantly changed only by Rapa + DR, e.g., 35% of the transcripts up-regulated and 49% of the transcripts down-regulated by Rapa + DR were not significantly changed by either DR or Rapa alone. Our analysis of specific genes altered by the combination of DR and Rapa showed that the combination of these two interventions enhances the effect of DR or Rapa alone by inducing the expression of genes that were not significantly altered by either one alone. When we scrutinized the list of genes of the Rapa + DR group and compared the list to either the DR or Rapa gene list, we observed that the majority of the genes that appear as new genes in the combined treatment were also altered in the same direction by DR or Rapa but did not reach statistical significance; only a handful of genes appears as a new genes [e.g., transcobalamine (Tcn2), sulfiredoxin1 (Srxn1), CD81 antigen (CD81)]. Based on these data, we propose that the combination of Rapa and DR has an additive effect on the expression of a large number of genes, which is supported by the heatmap data. The combination of Rapa and DR has not only a larger effect on gene expression (more genes become significant), but also a greater fold change in transcript levels and a greater fraction of the mice show an increase in the transcripts. With respect to the pathways altered by Rapa + DR, we also observed that more pathways were significantly changed by Rapa + DR than by either DR or Rapa alone. Six pathways were shared between DR and Rapa + DR in the top 15 pathways: mitochondrial dysfunction, glucocorticoid receptor signaling, the super pathway of melatonin degradation, melatonin degradation I, nicotine degradation II, and serotonin degradation. For all these pathways, most of the genes are up-regulated e.g., electron transport chain complexes, ATPase subunits, and NADH dehydrogenase for mitochondrial dysfunction pathway, suggesting that mitochondrial function might be improved. An improved glucocorticoid receptor signaling would suggest improved anti-inflammatory response (Smoak & Cidlowski, 2004). As for the melatonin degradation, nicotine degradation, and serotonin degradation, the majority of the changes in these pathways share components or isoforms of the cytochrome p450 family, which is an important group of enzymes responsible for drug metabolism and detoxification. These data are consistent with the reports showing that DR up-regulates drug metabolism in the liver (Sachan & Das, 1982). It is also interesting that genes involved in drug metabolism are up-regulated in livers of the long-lived Ames dwarf mouse (Boylston *et al*., 2006).

Of the 34 pathways that are altered only by Rapa, approximately 70% were down-regulated, e.g., l-serine and alanine degradation, glycolysis, and IL-15 production pathways were most affected. Our data are consistent with previous data showing that Rapa altered amino acid degradation (Beck *et al*., 1999). The decrease in the glycolysis pathway has been associated with the protective effects of Rapa against cancer, e.g., a down-regulation in glycolysis has been associated with decreased cell size and cell proliferation, two important factors for cancer progression (Edinger *et al*., 2003). IL-15 is a cytokine constitutively expressed by a large number of cell types and tissues (including monocytes, macrophages, dendritic cells, keratinocytes, fibroblasts and nerve cells) and plays an important role in innate and adaptive immunity. Decreased levels of this cytokine have been proposed to be the mechanism underlying the immunosuppressive effect (Lodolce *et al*., 2002). The oncostatin M (OSM) and acetate conversion to acetyl CoA pathways were the two pathways up-regulated by Rapa that showed the largest effect. The OSM pathway has been shown to protect against cancer because it inhibits cell proliferation and promotes cell detachment, increased epithelial apoptosis, and enhanced clearance of epithelial structures during the remodeling phase of mammary involution (Tiffen *et al*., 2008). The up-regulation of the acetate conversion to Acetyl-CoA pathway may play a role in side effects of Rapa on lipid metabolism and insulin insensitivity. For example, the increase in the transcripts for acetyl CoA synthetase 1 and 3 would be predicted increase the conversion of acetate to acetyl CoA, resulting in increased fatty acid synthesis. This combined with Rapa’s inhibition of the fat storage (Ma *et al*., 2007) could lead to an accumulation of free fatty acids that would lead to insulin resistance, which has been reported in rodents treated with Rapa (Lamming *et al*., 2012).

The metabolome comparison showed even less similarity between DR and Rapa treatment, with no metabolites significantly changed in Rapa. However, when Rapa is given to DR mice, we observe a large number of new metabolites. Thus, the combination of DR and Rapa showed greater effects on the metabolome than DR alone, agreeing with our transcriptiome data, indicating that a combination of Rapa and DR may have additive affects. Our analysis showed that most of the metabolite pathways that are significantly changed by DR are related to regulation of energy status, e.g., amino acid, carbohydrate, lipid, and energy (which included the Krebs cycle and oxidative phosphorylation). Because the initial studies in yeast suggest that TOR is a sensor of amino acids that becomes inactive upon depletion of amino acids (Hall, 1996), it is likely that a reduction in protein (amino acids) is a key factor in reduced mTOR signaling in DR (e.g., proteins represent 19% of the total components of the Purine diet; see Table S3). However, growth factors, e.g., IGF1 and insulin have also been shown to activate mTOR signaling (via PI3K and Akt), and DR has been shown to reduce circulating levels of IGF-1 and insulin in rodents (Dunn *et al*., 1997). Thus, DR could alter mTOR signaling through multiple mechanisms.

Two major observations arise from our study. First, our data show that DR and Rapa have quite different effects on the liver transcriptome and metabolome, demonstrating that Rapa is not simply a mimetic of DR. As our studies give only insight into what effect DR and Rapa have on the liver transcriptome and metabolome, preliminary unpublished data from our laboratory show that we observe even greater differences between DR and Rapa mice in the transcriptome of adipose tissue. Thus, we argue based on our limited data that it is likely that similar differences in the transcriptome will be found in other tissues of mice fed a DR-diet or Rapa. As our study demonstrates that DR and Rapa have different and unique effects on the transcriptome, the data do not allow us to determine whether the effect of DR and Rapa on lifespan occur through similar or different mechanism(s) because DR and Rapa have comparable effects on 20–34% of the genes and pathways, respectively. For example, the protein ubiquitination, mTOR signaling, mitochondrial function, and the Nrf2 pathways are altered by both DR and Rapa, and these pathways have been proposed to be important in aging.

The second major observation from this study is that DR mice given Rapa showed a significant change in a large number of transcripts and metabolites, which were not changed significantly by DR or Rapa alone. This observation is further supported by our heatmap data, which showed that combining DR and Rapa resulted in a larger number of transcripts that were up or down-regulated when compared with DR or Rapa alone. The transcriptome data suggest that Rapa potentiates the effects of DR on the genes whose expression is up-regulated in the Rapa + DR mice, e.g., almost 90% of the genes that are increased by Rapa + DR show trend of increased expression in DR mice (Table S4). On the other hand, only 35% of the genes whose expression was reduced significantly in the Rapa + DR mice showed a non-significant decrease in the DR mice; almost 60% of the genes significantly reduced in Rapa + DR mice showed a trend toward reduced expression in mice fed only Rapa.

These data suggest that feeding DR mice Rapa would increase lifespan over that observed in DR mice. Currently, there is no information on the effect of a combination of DR and Rapa on the lifespan of mice or any mammal. However, the reported studies with invertebrates have been contradictory. Rapa increases the lifespan of *Drosophila* fed a DR-diet (Bjedov *et al*., 2010); but, the lifespans of TOR mutant yeast and *C. elegans* are not increased further by DR (Kaeberlein *et al*., 2005). Thus, it will be important in the future to determine whether combining DR and Rapa will increase lifespan further than that observed with DR and Rapa alone.

## Experimental procedure

### Animals and feeding regiment

C57/BL6 male mice were purchased from The Jackson Laboratory (Bar Harbor, ME, USA). At 2 months of age, the mice were divided into four dietary regimens using Purina Mills Test Diet Control #1810306 (Purina Mills, St. Louis, MO, USA): mice fed AL, mice fed 40% of the diet consumed by AL mice (diet restriction; DR), mice fed AL diet supplemented with 14 ppm of rapamycin (Rapa), and mice fed DR diet supplemented with 14 ppm of rapamycin (Rapa + DR). The DR diet was calculated to 60% of the food consumed by the AL fed mice on a daily basis over a week and was given to the DR mice 3 pm daily. The Rapa was administered using microencapsulated Rapa as described Harrison *et al*. (2009). Mice were maintained on these dietary regimens until 8 months of age (6 months of treatment), at which time the mice were euthanized by carbon dioxide and liver tissues were harvested, snap-frozen in liquid nitrogen, and stored at −80°C until used. All procedures followed the guidelines approved by the Institutional Animal Care and Use Committee at the University of Texas Health Science Center at San Antonio.

### Microarray processing and analysis

Microarray processing was carried out at the National Institute on Aging, Gene Expression and Genomics Unit. RNA from frozen liver (25 mg), extracted from liver tissues (*N* = 11–12 mice; Supporting information) was then processed into cRNA probes for hybridization to arrays using Illumina Total RNA prep kit from Ambion (Live Technologies, Grand Island, NY, USA) following manufacturer’s protocols. Liver cRNA probes were then hybridized to Illumina Mouse Ref8 microarrays (V2.0; Illumina, San Diego, CA) following manufacturer’s protocol and arrays were scanned by iSCAN system (Illumina). iDAT data outputs from the iSCAN system were extracted by Genome Studios software (v 1.6; Illumina). Data were then transformed and normalized using log_2_ transformation and the z-normalization method as described by Cheadle *et al*. (2003). For statistical analysis, microarray data were processed using r (v 2.15.1; R Development Core Team, Vienna, Austria) using one-way ANOVA with pairwise comparisons. Multiple testing corrections were then applied to the data set using r package ‘*q* value’ (v 1.30.0, Dabney, A and Storey, J) to control for FDR. Principal component analysis was done using jmp genomics 5 (SAS, Cary, NC, USA). We identified differential expressions between treatment conditions using a FDR cutoff of *q* value < 0.05 in DR vs. AL, Rapa vs. AL, and RAPA + DR vs. AL, and had a median fold change > 15%. Further data analysis was done with the following comparisons: Rapa vs. DR and RAPA + DR vs. DR. Genes considered significantly changed were plotted in heatmap with average linkage hierarchical cluster and Euclidean distance using Matlab (2011a; The Mathworks, Natick, MA, USA). Pathway analyses were carried out using Ingenuity Pathway Analysis (Ingenuity Systems, Redwood City, CA, USA), which provided association of significantly changed genes into pathways.

### Metabolomics

Frozen liver tissues obtained from DR (*N* = 7), Rapa + DR (*N* = 9), AL (*N* = 10) and Rapa (*N* = 10) mice were analyzed for metabolite levels by Metabolon (Durham, NC, USA) using GC/MS and LC/MS/MS to identify metabolites using a library of over 1000 compounds and quantify the levels of the metabolites in the samples. Data were normalized by Metabolon and statistical analysis was done using ANOVA with pairwise comparisons. False discovery analysis was then applied to the dataset using r package ‘*q* value’ (v 1.30.0, Dabney, A and Storey, J). Metabolon imputed the data for metabolites that had samples that were not detected using the lowest detected data value available for that metabolite. We excluded all metabolites that had imputed value > 20% in the groups we analyzed for the pairwise comparisons. Statistical significance was indicated at *q*-value < 0.05 and fold change > 15%.

## Data deposition

Microarray data for this study has been deposited in Gene Expression Omnibus (GEO; GSE40977) and can be viewed at: http://www.ncbi.nlm.nih.gov/geo/query/acc.cgi?acc=GSE40977.

## Supporting Information

**Data S1** Methods.

**Fig. S1** mTOR signaling pathways have similar decrease in all three groups. **Fig. S2** Rapamycin levels in livers of Rapa and Rapa + DR mice. **Fig. S3** qRT-PCR of genes in the livers of female mice fed multiple doses of rapamycin. **Table S1** Both linear discrimination predictor and quadratic discriminant analysis show differences between Rapa and DR. **Table S2** Percent change in the transcriptome analysis using various filtering criteria. **Table S3** Caloric and diet composition of LabDiet 5LG6-JL diet. **Table S4** Genes potentiated by the combined treatment of rapamycin and dietary restriction. **File S1** File (SF1- Dataset.xls) containing data for significantly changed gene list, IPA pathways, pathway comparisons, and metabolites.
