# Supplementary material for: Combined treatment of rapamycin and dietary restriction has a larger effect on the transcriptome and metabolome of liver
Source: Aging Cell. 2013 Dec 5;13(2):311–9. doi: 10.1111/acel.12175 (PMC3989927; doi:10.1111/acel.12175)
Supplement: Supplementary file 1 — Data S1 Methods. [file acel0013-0311-sd1.doc]

**Supplementary Methods:**

*mTOR Signaling*

Frozen liver tissue was homogenized in RIPA buffer (1× PBS, 1% Nonidet P-40, 0.5% sodium deoxycholate, 0.1% SDS) supplemented with protease and phosphatase inhibitors (Roche, Indiana) using a Dounce homogenizer. Following homogenization, the homogenate was centrifuged at 4 °C, 12,000 x g for 10 min and the supernatant was collected. Samples were resolved by SDS–PAGE and followed by transfer to PDVF membrane. Western blot analysis was done using antibodies against the targeted proteins of interest: actin, S6 and phospho-S6 (Ser235/236) (Cell Signaling, Danvers, MA.). The ratio of phosopho-S6 levels to total S6 levels was used to assess mTOR signaling.

*Rapamycin levels in Liver tissue*

Rapamycin levels were measured in liver tissue by a HPLC system coupled to an AB Sciex API 3200 tandem mass spectrometer with turbo ion spray. Rapa and ASCO (internal standard) obtained from LC Laboratories (Woburn, MA) were used in the quantification of Rapa. Approximately 100 mg of samples and 0.1 mL of calibrator were mixed by sonification with 10 µL of 0.5 µg/mL ASCO and mixed with a solution containing 0.1% formic acid and 10 mM ammonium formate dissolved in 95% HPLC grade methanol. The samples were then vortexed and centrifuged at 15,000 *g* for 5 min at 23°C. Supernatants were then injected into the LC/MS/MS. The ratio of the peak area of Rapa to that of the internal standard ASCO (response ratio) for each unknown sample was compared against a standard curve using different concentrations of Rapa standard. The concentration of Rapa was expressed as pg/mg of liver tissue protein.

*RNA processing*

RNA from frozen liver (25 mg) was extracted from liver tissues (N=11 to 12 mice) using RNeasy kit (Qiagen, Valencia, CA) following manufacturer’s protocols. RNA concentration was determined by Nanodrop (Thermo Scientific, Wilmington, DE) and RNA quality was determined by agarose gel and Agilent Bioanalyzer (Agilent Technologies, Santa Clara, CA).

*Quantitative Real Time PCR (qRT-PCR)*

Total RNA was extracted from frozen liver tissues (25mg) using the RNeasy kit (Qiagen, Valencia, CA) according to the manufacturer’s instructions and DNA contamination was removed by Turbofree DNase (Ambion, Inc., Foster City, CA). The RNA yield of each sample was determined spectrophotometrically, assuming that 1 optical density at 260 nm (OD_260_) unit = 40 mg/L. The quality of total RNA extracted from each sample was monitored by A260:A280 ratio and 1.0% agarose formaldehyde gel electrophoresis. One µg of RNA was used to generate cDNA using the Retroscript kit (Ambion, Inc., Foster City, CA). Primers were designed using Primer Express (Applied Biosystem, Inc., Foster City, CA) and Primer-BLAST (NCBI). Quantitative real-time RT-PCR (qRT-PCR) was performed using SYBR Green PCR Master Mix (Applied Biosystem, Inc., Foster City, CA) in a 96 well plate using *Gapdh* as a housekeeping control with detection by a 7500 Real-Time PCR Detection System (Applied Biosystem, Inc., Foster City, CA).

***Supplementary Figure 1: mTOR signaling pathways have similar decrease in all three groups.***

mTOR signaling was measured as pS6/total S6 in the liver of AL, Rapa, DR, and Rapa+DR mice as described by Fok, WC et al (2012). Representative western blot measuring phoso-S6, Total S6, and actin as a loading control are shown **(A)**. The pS6/total S6 levels were measured from the western blots and shown as a box and whisker plot, where the mean is the horizontal line in the box, the upper half of the box indicates the 75% quantile, while the lower half of the box indicates the 25% quantile **(B)**. The top bar indicates the maximum value and the bottom bar indicates the minimum value. The data were collected from 6 samples per group and was analyzed by one-way ANOVA followed by tukey post-hoc test. Significant p-values for the following pairwise comparisons are listed: p=0.0487 between Rapa and AL, p<0.001 between DR and AL, and p<0.001 between Rapa+DR and AL.

***Supplementary Figure 2: Rapamycin Levels in Livers of Rapa and Rapa+DR Mice***

Rapamycin levels were measured in the livers of Rapa (n = 23) and Rapa+DR (n = 25) mice, and the data are expressed as Rapa levels (pg) per mg of liver protein. The mean is marked with a red horizontal line and the bars indicate the standard error of the mean. The mean ± SEM for Rapa mice is 14.1 ± 0.9 and for Rapa+DR mice is 9.7 ± 1.0, and this difference is significant at the p=0.002 level.

***Supplementary Table 1: Both Linear discrimination predictor and Quadratic Discriminant Analysis show differences between Rapa and DR.***

The statistical comparison of the top 3 principal component was calculated using a Linear discrimination predictor **(A).** For the linear discrimination predictor, and AUC of 0.5 indicates no separation between groups), an AUC of 1 (perfect separation) between groups with the 95% confidence interval shown in parentheses: Rapa vs. AL, AUC of 0.8 (0.58,1.02); Rapa vs. DR and DR vs. AL, AUC of 1 (1,1); Rapa+DR vs. AL, AUC of 0.96 (0.88, 1.04); Rapa+DR vs. DR, AUC of 0.77 (0.56,0.97); and Rapa+DR vs. Rapa, AUC of 0.95 (0.85, 1.05). The parenthesis indicates a 95% confidence interval for each value. Quadratic discriminant analysis (B) with groups indicated at the top row and in parentheses are the total possible samples for that group. The rows indicate the prediction from the analysis and in parentheses is the percentage of the predicted against total known.

***Supplementary Table 2: Percent change in the transcriptome analysis using various filtering criteria.***

Percentages of each respective bin of the venn diagram representation of the transcriptome analysis for filtering criteria of q<0.05 and 15%, 20%, or 30% change (Figure 2A-F) in DR vs. Rapa only for genes up-regulated **(A)** and down-regulated **(B)** and in the comparison of DR, Rapa, and Rapa+DR for genes up-regulated **(C)** and down-regulated **(D)**.

***Supplementary Figure 3: qRT-PCR of genes in the livers of female mice fed multiple doses of Rapamycin***

Gene expression was determined by qRT-PCR on representative genes for the following pathways: **(A)** ubiquitin pathway: *Flot1*, *Psmd9;* **(B)** circadian rhythm: *Clock, Bmal; and* **(C)** unfolding protein response: *Hspa9*, *Pdia4;* were determined in the livers of AL (solid bars), DR (open bars), and Rapa at different doses (gray bars) mice. The data were obtained from 8 female mice per group and expressed as mean ± SEM. Data were analyzed using one-way ANOVA with the Tukey’s post-hoc test; an asterisk denotes those values that are significantly different (p≤0.05) from AL mice.

***Supplementary Table 3: Caloric and Diet Composition of LabDiet 5LG6-JL Diet.***

Diet composition is listed in percent of calories from carbohydrates, proteins, and fat **(A)** and percent composition of dietary carbohydrates, proteins, and fat **(B)**. This data was simplified from the Labdiet 5LG6-JL datasheet.

***Supplementary Table 4: Genes Potentiated by the Combined Treatment of Rapamycin and Dietary Restriction***

The percent of genes that were potentiated by Rapa+DR treatment but did not reach statistical significance in DR or Rapa treatments are shown. The data was obtained from the gene list (gene expression data set) considering the total number of genes significantly up or down-regulated only by DR+Rapa as 100% respectively.

***Supplementary File 1: File (SF1- Dataset.xls) containing data for significantly changed gene list, IPA pathways, pathway comparisons, and metabolites.***

**Tab1 (“Genes”):** A list of all genes significantly different (using criteria of q<0.05 and>15% change median) from AL is given in the same order from top to bottom as the gene hierarchical cluster from heatmap in Figure 3A. The group column indicates where the genes are found in the Venn diagram (Figure 2A and 2B). The genes found significant with filtering criteria of >30% change with q<0.05 and >15% change with q<0.001 (Figure 2C-2E) are shown in the “Significant in” columns. The median fold change is highlighted with red indicating significantly up-regulated genes, green for significantly down-regulated genes, and white for non-significant genes.

**Tab2 (“IPA-DR|AL”):** IPA pathways for genes significant in DR/AL with a p<0.05 in the Fisher’s exact test, ranked from lowest to the highest p-value.

**Tab3 (“IPA-Rapa|AL”):** IPA pathways for genes significant in Rapa/AL with a p<0.05 in the Fisher’s exact test, ranked from lowest to the highest p-value.

**Tab4 (“IPA-RDR|AL”):** IPA pathways for genes significant in Rapa+DR/AL with a p<0.05 in the Fisher’s exact test, ranked from lowest to the highest p-value.

**Tab5: (“Pathway Comparison”):** All the pathways that were analyzed by IPA in DR, Rapa, and Rapa+DR are shown together with * indicating pathways that are significantly changed at p<0.05.

**Tab6: (“Metabolites”):** A list of metabolites significantly different (using criteria of q<0.05 and >15% change) from AL is sorted from top to bottom in the order of Figure 5C and 5D.  The group column indicates where the metabolites are located in the venn diagram (Figure 5A and 5B).  The means of the data are highlighted with red indicating metabolites significantly increased, green for metabolites significantly decreased, and white for metabolites whose levels do not change significantly.
