# Supplementary material for: Combined treatment of rapamycin and dietary restriction has a larger effect on the transcriptome and metabolome of liver
Source: Aging Cell. 2013 Dec 5;13(2):311–9. doi: 10.1111/acel.12175 (PMC3989927; doi:10.1111/acel.12175)
Supplement: Supplementary file 2 — Fig. S1 mTOR signaling pathways have similar decrease in all three groups. Fig. S2 Rapamycin levels in livers of Rapa and Rapa + DR mice. Fig. S3 qRT-PCR of genes in the livers of female mice fed multiple doses of rapamycin. Table S1 Both linear discrimination predictor and quadratic discriminant analysis show differences between Rapa and DR. Table S2 Percent change in the transcriptome analysis using various filtering criteria. Table S3 Caloric and diet composition of LabDiet 5LG6-JL diet. Table S4 Genes potentiated by the combined treatment of rapamycin and dietary restriction. File S1 File (SF1- Dataset.xls) containing data for significantly changed gene list, IPA pathways, pathway comparisons, and metabolites. [file acel0013-0311-sd2.pptx]

## Slide 1
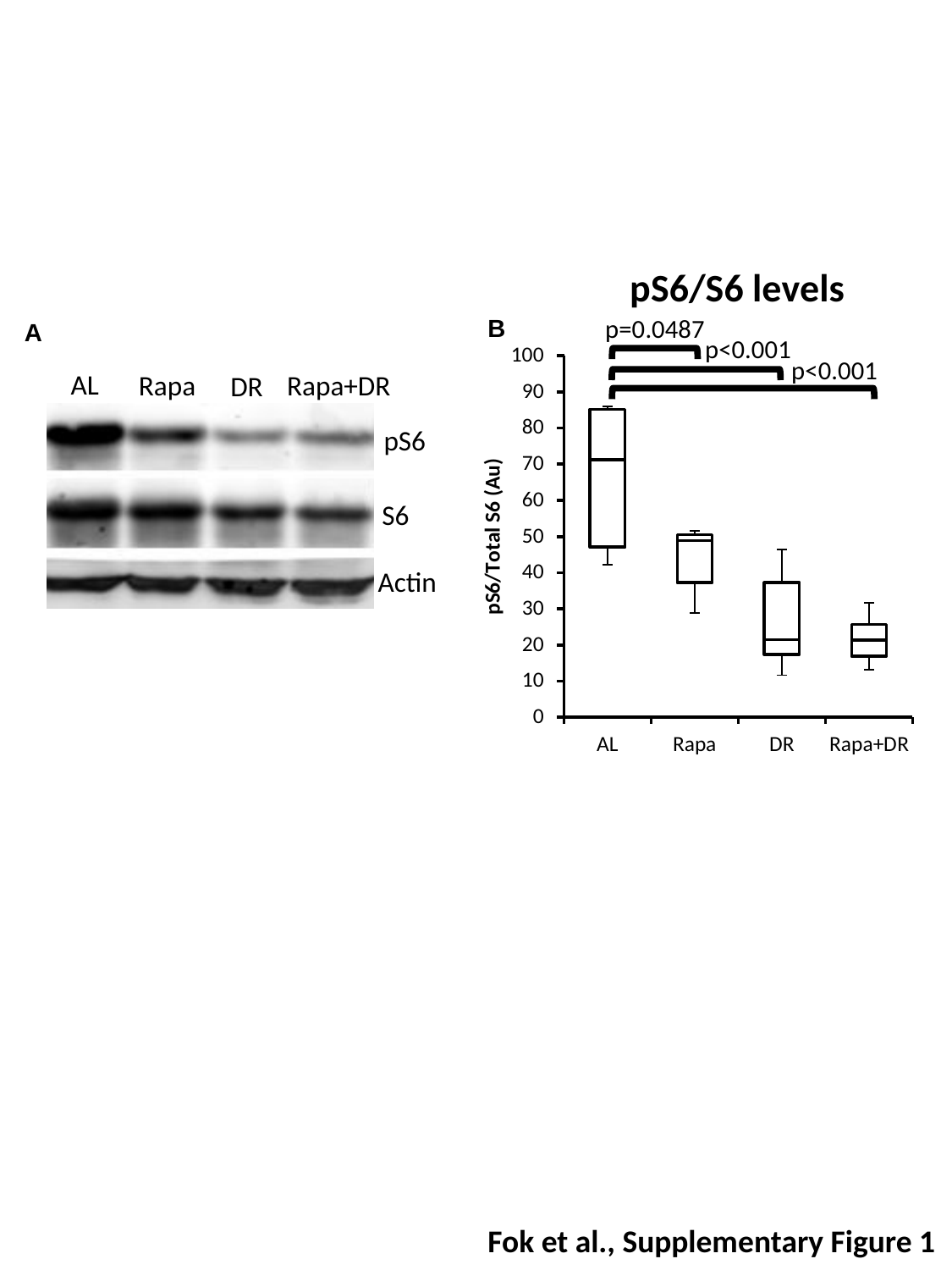

B
A
AL
Rapa+DR
Rapa
DR
pS6
S6
Actin
Fok et al., Supplementary Figure 1

## Slide 2
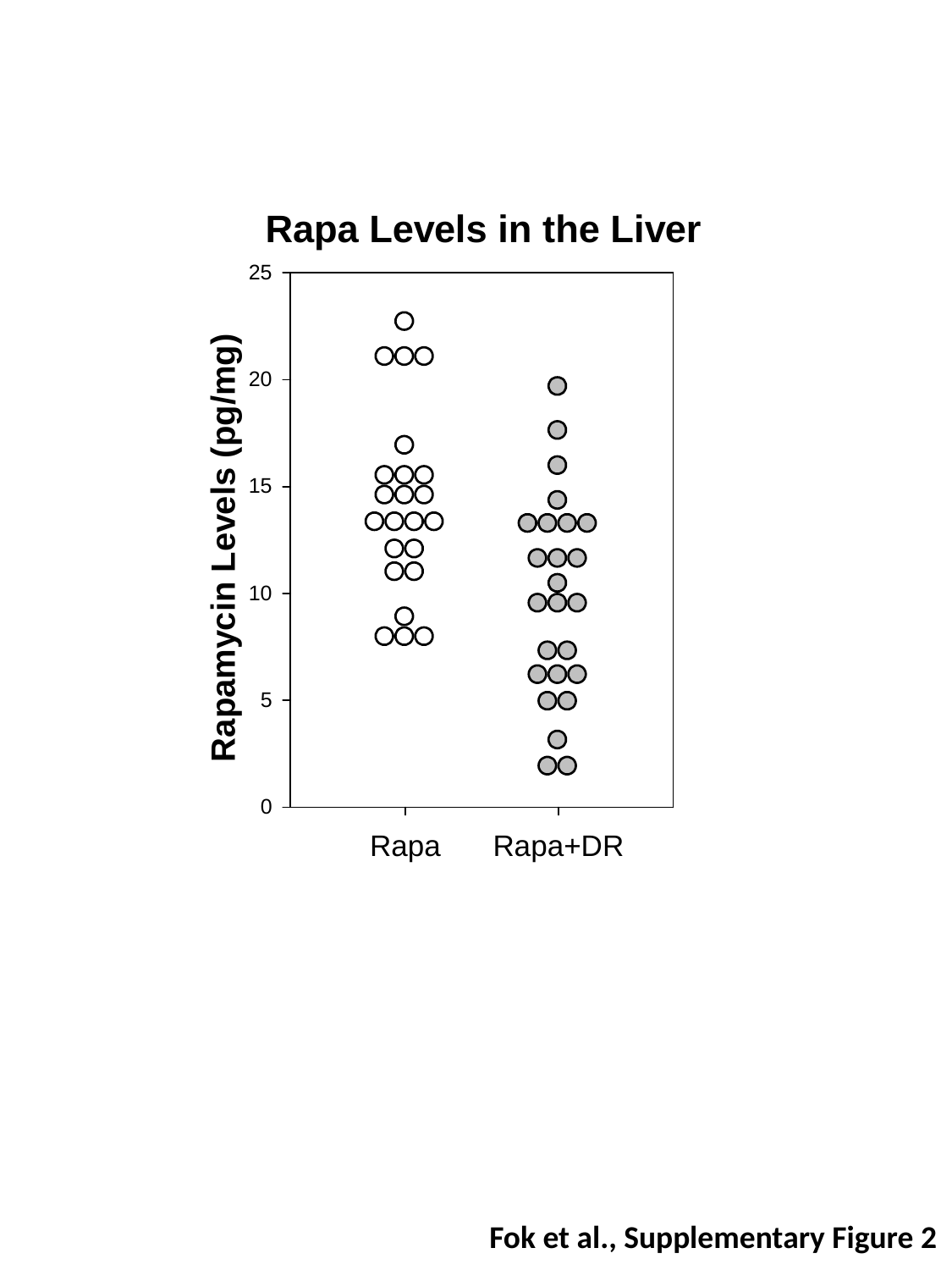

Fok et al., Supplementary Figure 2

## Slide 3
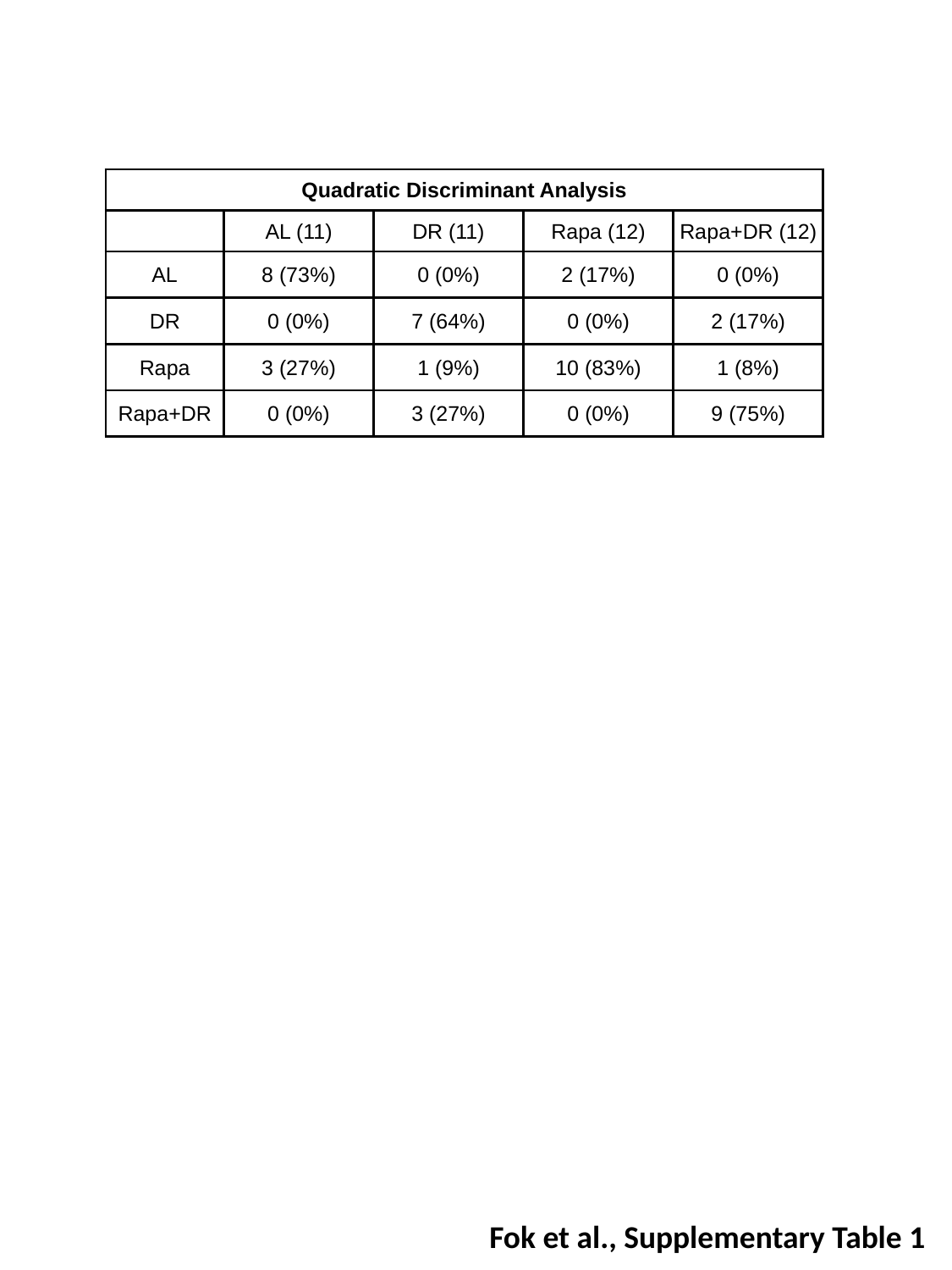

| Quadratic Discriminant Analysis | | | | |
| --- | --- | --- | --- | --- |
| | AL (11) | DR (11) | Rapa (12) | Rapa+DR (12) |
| AL | 8 (73%) | 0 (0%) | 2 (17%) | 0 (0%) |
| DR | 0 (0%) | 7 (64%) | 0 (0%) | 2 (17%) |
| Rapa | 3 (27%) | 1 (9%) | 10 (83%) | 1 (8%) |
| Rapa+DR | 0 (0%) | 3 (27%) | 0 (0%) | 9 (75%) |
Fok et al., Supplementary Table 1

## Slide 4
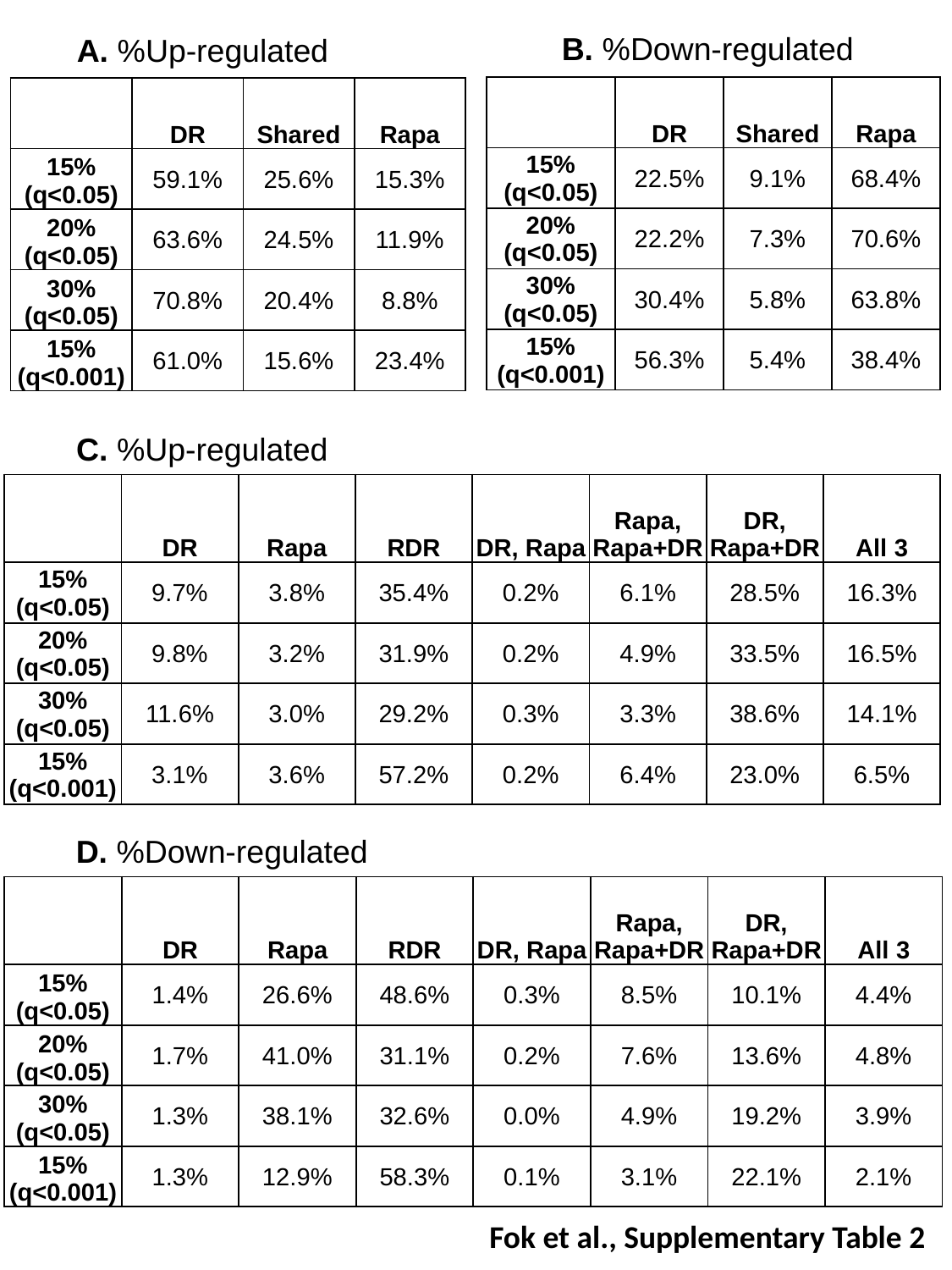

B. %Down-regulated
A. %Up-regulated
| | DR | Shared | Rapa |
| --- | --- | --- | --- |
| 15% (q<0.05) | 22.5% | 9.1% | 68.4% |
| 20% (q<0.05) | 22.2% | 7.3% | 70.6% |
| 30% (q<0.05) | 30.4% | 5.8% | 63.8% |
| 15% (q<0.001) | 56.3% | 5.4% | 38.4% |
| | DR | Shared | Rapa |
| --- | --- | --- | --- |
| 15% (q<0.05) | 59.1% | 25.6% | 15.3% |
| 20% (q<0.05) | 63.6% | 24.5% | 11.9% |
| 30% (q<0.05) | 70.8% | 20.4% | 8.8% |
| 15% (q<0.001) | 61.0% | 15.6% | 23.4% |
C. %Up-regulated
| | DR | Rapa | RDR | DR, Rapa | Rapa, Rapa+DR | DR, Rapa+DR | All 3 |
| --- | --- | --- | --- | --- | --- | --- | --- |
| 15% (q<0.05) | 9.7% | 3.8% | 35.4% | 0.2% | 6.1% | 28.5% | 16.3% |
| 20% (q<0.05) | 9.8% | 3.2% | 31.9% | 0.2% | 4.9% | 33.5% | 16.5% |
| 30% (q<0.05) | 11.6% | 3.0% | 29.2% | 0.3% | 3.3% | 38.6% | 14.1% |
| 15% (q<0.001) | 3.1% | 3.6% | 57.2% | 0.2% | 6.4% | 23.0% | 6.5% |
D. %Down-regulated
| | DR | Rapa | RDR | DR, Rapa | Rapa, Rapa+DR | DR, Rapa+DR | All 3 |
| --- | --- | --- | --- | --- | --- | --- | --- |
| 15% (q<0.05) | 1.4% | 26.6% | 48.6% | 0.3% | 8.5% | 10.1% | 4.4% |
| 20% (q<0.05) | 1.7% | 41.0% | 31.1% | 0.2% | 7.6% | 13.6% | 4.8% |
| 30% (q<0.05) | 1.3% | 38.1% | 32.6% | 0.0% | 4.9% | 19.2% | 3.9% |
| 15% (q<0.001) | 1.3% | 12.9% | 58.3% | 0.1% | 3.1% | 22.1% | 2.1% |
Fok et al., Supplementary Table 2

## Slide 5
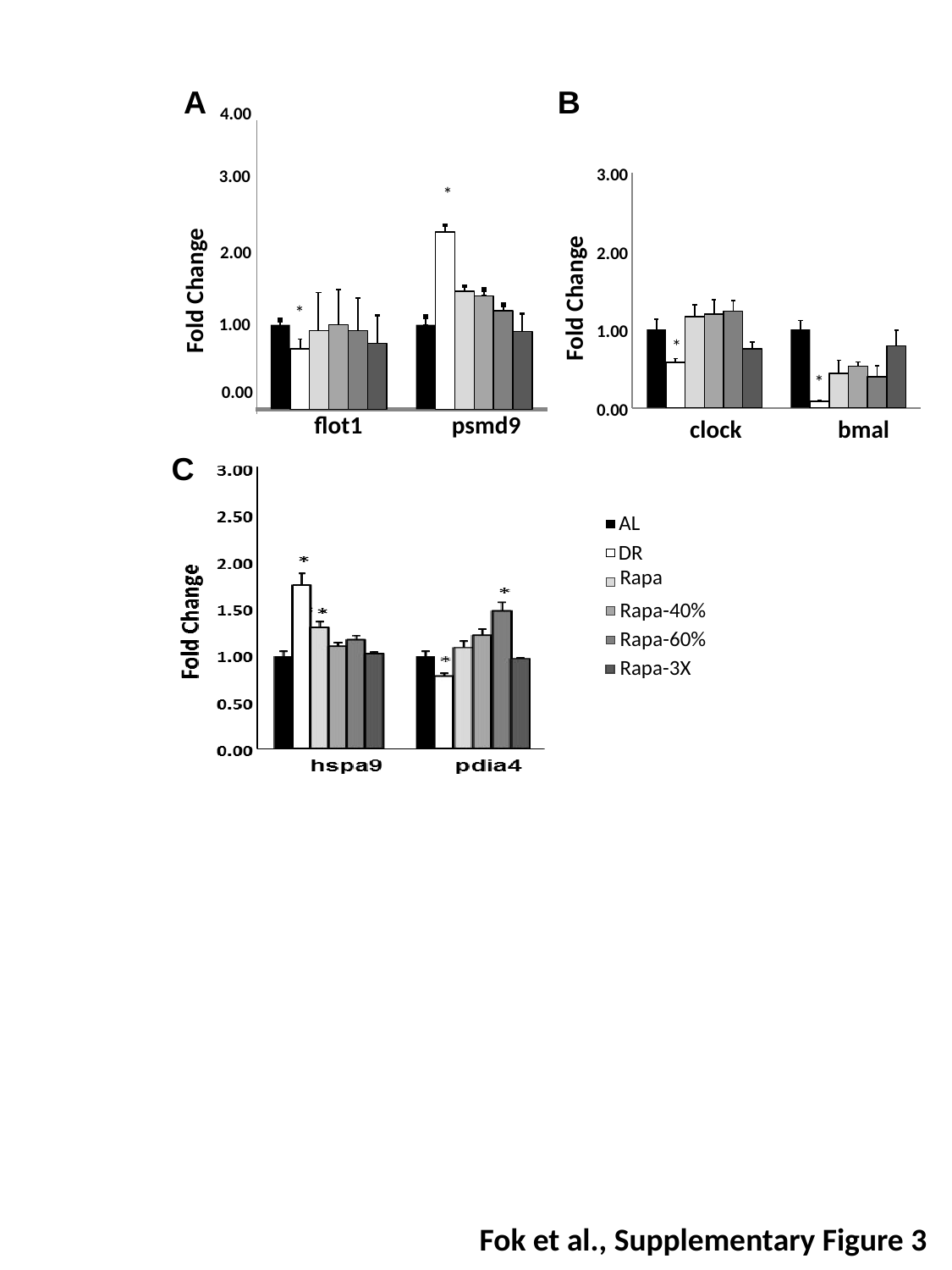

A
B
4.00
*
*
3.00
Fold Change
2.00
1.00
0.00
flot1
psmd9
3.00
2.00
Fold Change
1.00
*
*
0.00
clock
bmal
C
AL
DR
Rapa
Rapa-40%
Rapa-60%
Rapa-3X
Fok et al., Supplementary Figure 3

## Slide 6
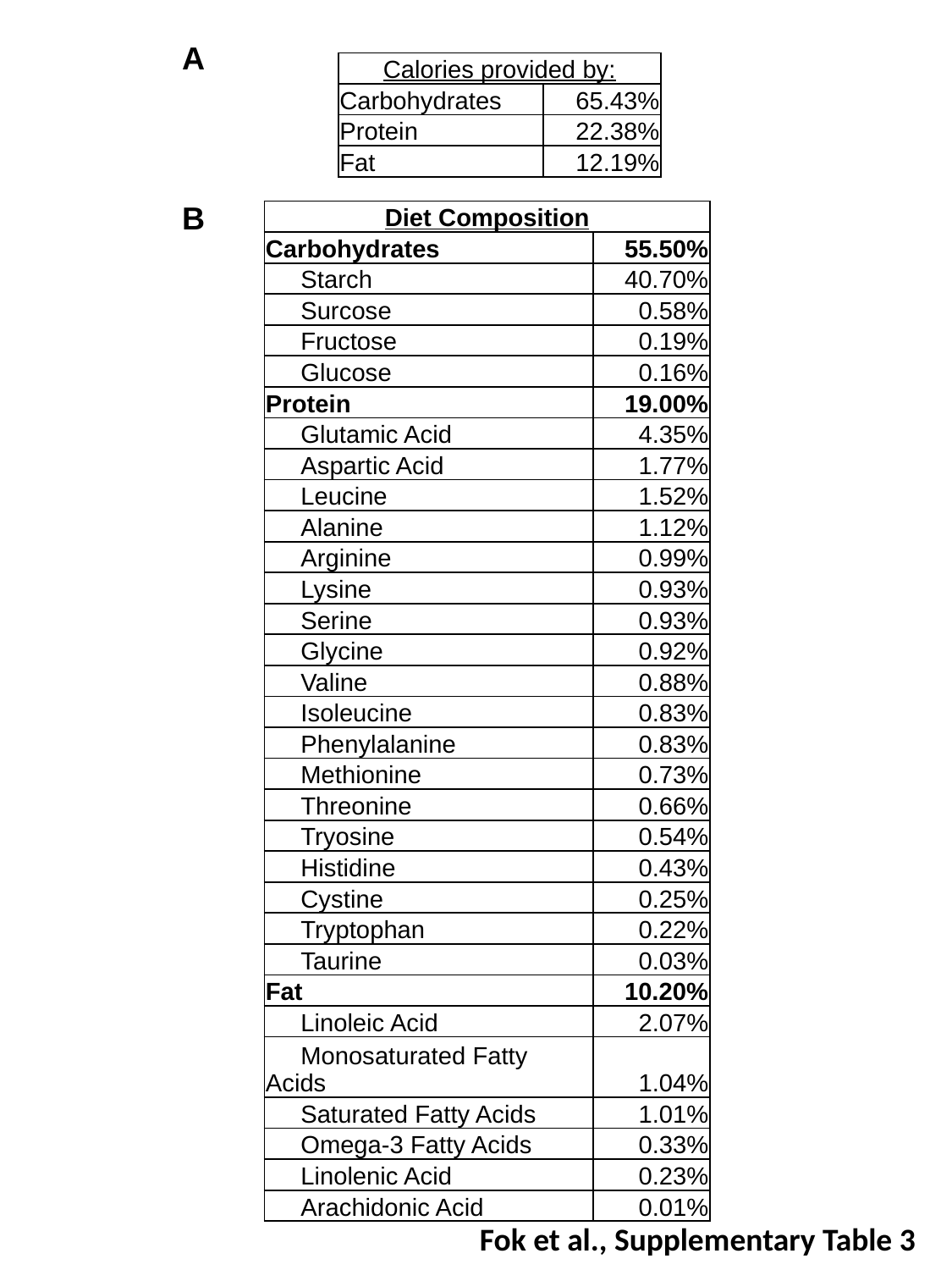

A
| Calories provided by: | |
| --- | --- |
| Carbohydrates | 65.43% |
| Protein | 22.38% |
| Fat | 12.19% |
B
| Diet Composition | |
| --- | --- |
| Carbohydrates | 55.50% |
| Starch | 40.70% |
| Surcose | 0.58% |
| Fructose | 0.19% |
| Glucose | 0.16% |
| Protein | 19.00% |
| Glutamic Acid | 4.35% |
| Aspartic Acid | 1.77% |
| Leucine | 1.52% |
| Alanine | 1.12% |
| Arginine | 0.99% |
| Lysine | 0.93% |
| Serine | 0.93% |
| Glycine | 0.92% |
| Valine | 0.88% |
| Isoleucine | 0.83% |
| Phenylalanine | 0.83% |
| Methionine | 0.73% |
| Threonine | 0.66% |
| Tryosine | 0.54% |
| Histidine | 0.43% |
| Cystine | 0.25% |
| Tryptophan | 0.22% |
| Taurine | 0.03% |
| Fat | 10.20% |
| Linoleic Acid | 2.07% |
| Monosaturated Fatty Acids | 1.04% |
| Saturated Fatty Acids | 1.01% |
| Omega-3 Fatty Acids | 0.33% |
| Linolenic Acid | 0.23% |
| Arachidonic Acid | 0.01% |
Fok et al., Supplementary Table 3

## Slide 7
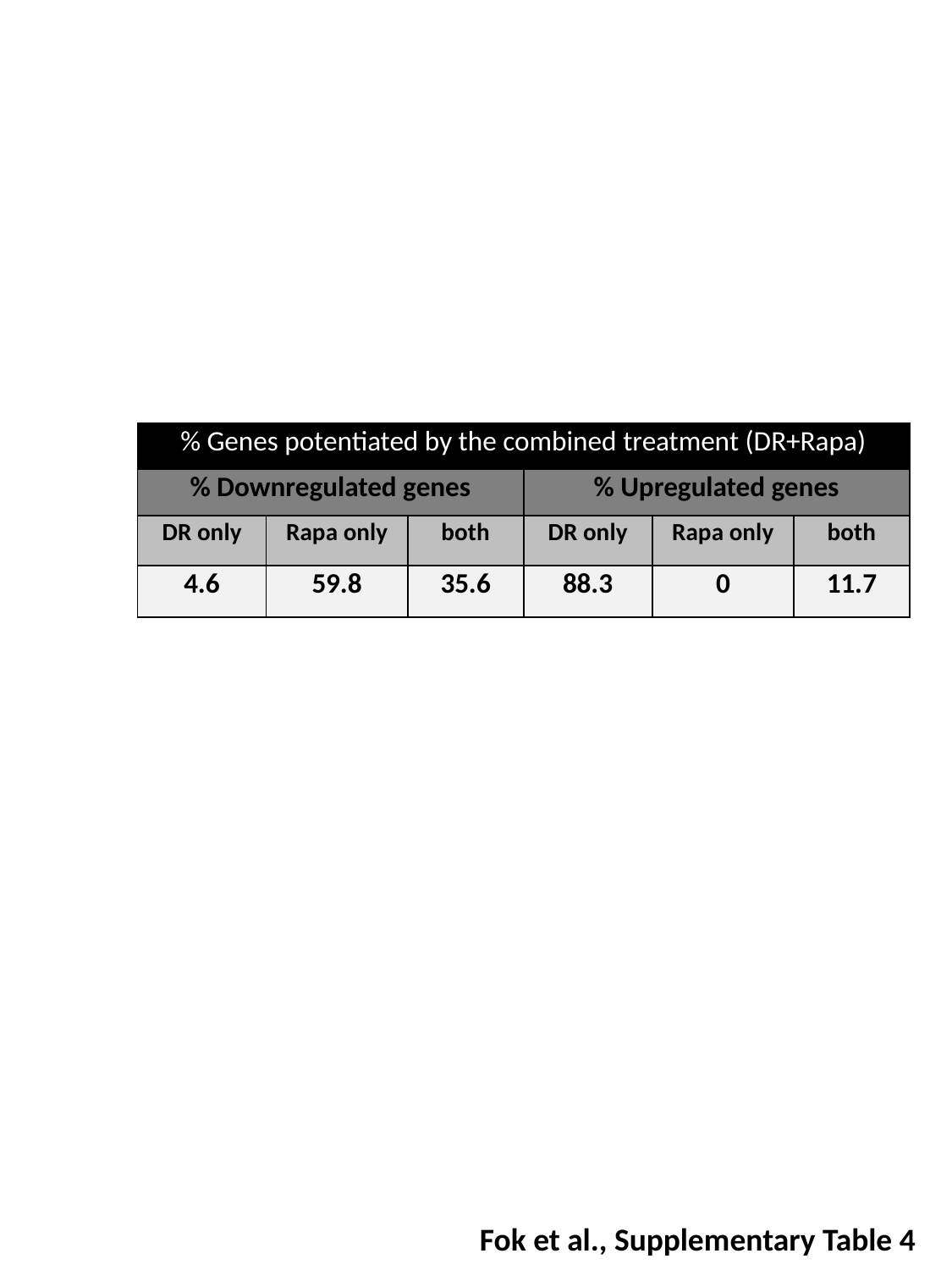

| % Genes potentiated by the combined treatment (DR+Rapa) | | | | | |
| --- | --- | --- | --- | --- | --- |
| % Downregulated genes | | | % Upregulated genes | | |
| DR only | Rapa only | both | DR only | Rapa only | both |
| 4.6 | 59.8 | 35.6 | 88.3 | 0 | 11.7 |
Fok et al., Supplementary Table 4

## Slide 8
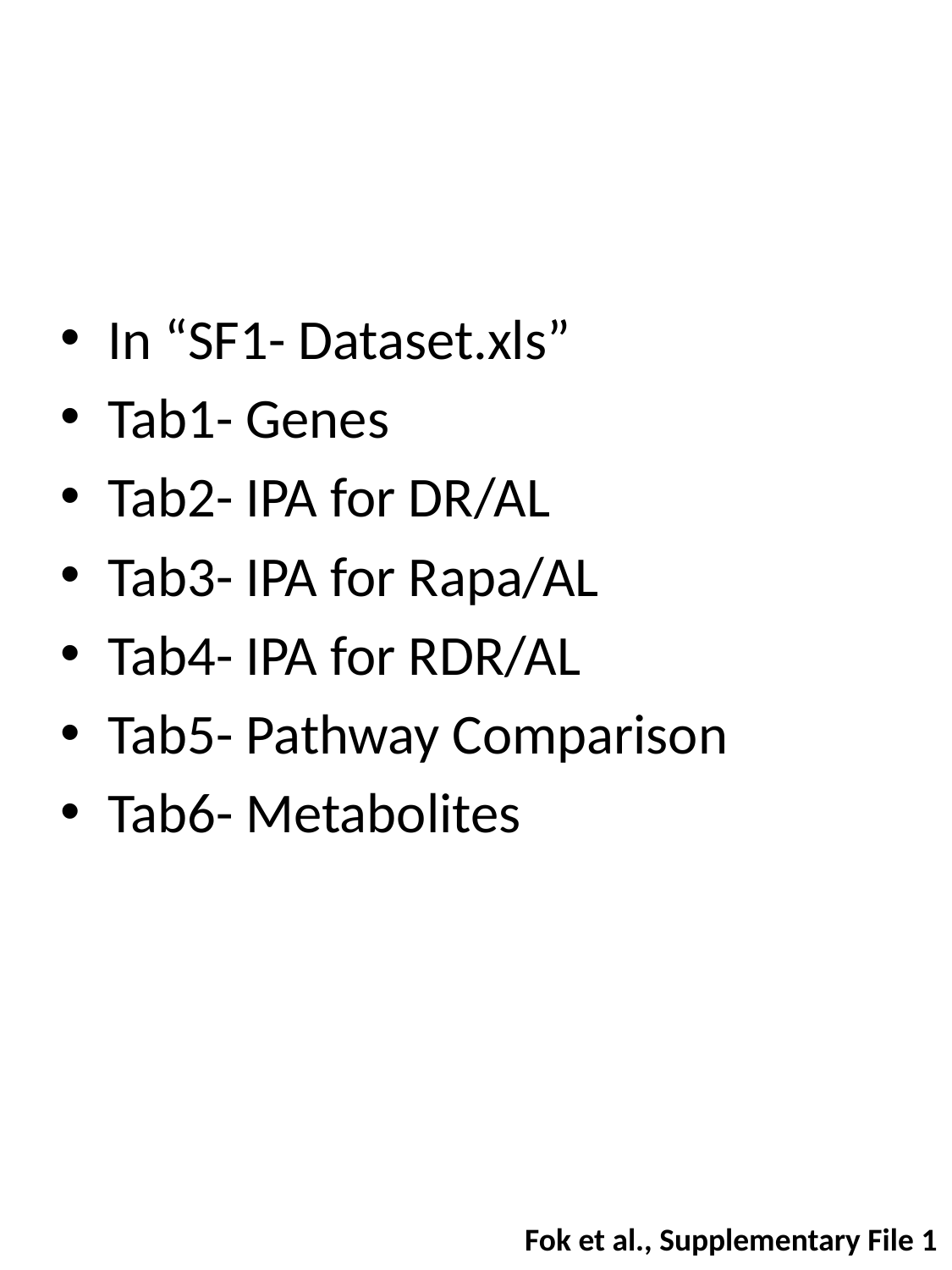

In “SF1- Dataset.xls”
Tab1- Genes
Tab2- IPA for DR/AL
Tab3- IPA for Rapa/AL
Tab4- IPA for RDR/AL
Tab5- Pathway Comparison
Tab6- Metabolites
Fok et al., Supplementary File 1
